# Low Systolic Blood Pressure and Mortality From All-Cause and Vascular Diseases Among the Rural Elderly in Korea; Kangwha Cohort Study

**DOI:** 10.1097/MD.0000000000000245

**Published:** 2015-01-16

**Authors:** Sang-Wook Yi, Seri Hong, Heechoul Ohrr

**Affiliations:** From the Department of Preventive Medicine and Public Health, Catholic Kwandong University, College of Medicine, Gangneung, Gangwon-do (S-WY); Department of Preventive Medicine (SH); and Institute for Health Promotion, Graduate School of Public Health, Yonsei University, Seoul, Republic of Korea (HO); Department of Preventive Medicine, Yonsei University College of Medicine (HO).

## Abstract

The association between low systolic blood pressure (SBP) and vascular diseases is unclear. The aim of this study was to prospectively examine the association between SBP, especially low SBP, and mortality from all causes and vascular diseases among the elderly in Korea.

Six thousand two hundred ninety four residents in a rural community were followed-up for deaths from 1985 to 2008. The adjusted hazard ratios (aHR) and 95% confidence intervals were calculated by Cox proportional hazard model. A stratified analysis was conducted by age at enrolment.

Among the elderly aged 65 and above, the lowest SBP (<100 mm Hg) group had an elevated aHR for mortality from vascular diseases (aHR = 2.1, 95% CI = 1.2–3.9) including stroke (aHR = 2.4, 95% CI = 0.9–6.3) and ischemic heart diseases (aHR = 5.1, 95% CI = 1.0–26.0) compared to those with SBP of 100–119 mm Hg, while higher SBP was associated with higher mortality. This J-curve association was generally maintained when analysis was restricted to those with fair or good self-rated health, or those with no known vascular diseases. In people below 65, increasing SBP nearly monotonically increased the mortality from all-cause and vascular diseases.

Our results suggest that elderly persons with low SBP should be treated with caution, since low SBP may increase vascular mortality.

## Abstract 2

Supplemental Digital Content is available in the text

## INTRODUCTION

Hypertension is a well-known risk factor for all-cause and vascular mortality.^1^ Meanwhile, several studies have reported a J- or U-shape association between blood pressure (BP) and vascular mortality, and have suggested that lower BP may increase the morbidity and mortality from vascular diseases, mainly among people with vascular or other diseases.^2–9^ In fact, J-curve association between BP and mortality should exist because below a certain point (a potential threshold), it will be difficult to sustain adequate perfusion to vital organs including heart and brain. However, the evidence for this association of low BP with vascular mortality, not to mention of the causality, is inconclusive, especially for low systolic blood pressure (SBP) in the general population,^1,3,10,11^ partly because the association of low SBP such as below 90 to 100 mm Hg with vascular mortality has rarely been examined in previous research.^1^

The aim of this study was thus to prospectively examine the association between SBP, especially low SBP, and mortality from all causes and vascular diseases among the elderly in a rural community. Since the participants were over 55 years of age, we focused on SBP, which has been shown to be a superior predictor of risk for vascular diseases compared to diastolic BP in the elderly.^5,12–14^

## METHODS

### Study Participants

This study used data from the Kangwha Cohort Study.^13,15^ Among 9378 residents of Kangwha county who were 55 years or older in February 1985, 6372 (67.9%) participated in interviews assessing their health behaviors, and their BP, height, and weight were also measured. Those with missing information on BP at entry (n = 39) or who were not followed up after the initial survey (n = 39), were excluded. As such, a final 6294 participants (2694 men, 3600 women) were included in the analysis. The Institutional Review Board of Human Research of Yonsei University approved the study.

### Data Collection

The primary survey was conducted in March 1985. Each participant was interviewed using a structured questionnaire including smoking status, drinking status, occupation, education, marital status, self-reported health, pre-existing hypertension, and pre-existing chronic diseases. BP was measured in a seated position by a trained investigator using a standard mercury sphygmomanometer. BP measurement training was provided with an educational audiotape produced by the London School of Hygiene & Tropical Medicine; the interobserver error in BP measurement was within 2 mm Hg.^13,15^ SBP was measured to the nearest 2 mm Hg as the first Korotkoff sounds. BP was measured once per person. Weight and height were determined with participants wearing light clothing. More details on the data collection can be found elsewhere.^15,16^

### Follow-Up and Outcome Ascertainment

Deaths among subjects from January 1, 1992 through December 31, 2008 were confirmed by the death records held at the National Statistical Office.^15^ Follow-up was performed through record linkage at the national level and was complete, except in the case of emigrants. Data for those who died from March 1985 through December 31, 1991 were collected either through calls, through visits from trained surveyors twice a year or from the burial and death certificates held at *eup* and *myeon* offices, which are administrative branch offices of local governments in Korea. The main outcomes for this study were death from all causes, vascular diseases (I00–I99), stroke (I60–I64), and ischemic heart diseases (I20–I25) as defined by the International Classification of Disease, Tenth Revision (ICD-10).

### Statistical Analysis

SBP was classified into 6 groups (mm Hg; <100, 100–119, 120–139, 140–159, 160–179, ≥180).^17,18^ In more detailed analysis, the lower end of SBP was further divided into 2 versions of 2 groups (mm Hg; <90, 90–99; <80, 80–99). Chi-squared tests and one-way analysis of variance (ANOVA) were performed to compare differences between the groups.

Cox proportional hazards models were used to evaluate the association between the baseline SBP and mortality. Analyses were adjusted for the following covariates: age at entry (continuous); sex; known hypertension (based on self-reported information: on regular medication, on irregular or no medication, no hypertension); smoking status (never smoked, former smoker, current smoker); drinking status (current drinker, non-drinker); occupation (agriculture, other); education (none, elementary school, middle school or above); marital status (living with, without spouse); self-reported health (good or fair, poor), and body mass index (BMI, kg/m^2^; <18.5, 18.5–20.9, 21.0–24.9, 25.0–27.4, ≥27.5). In the multivariate-adjusted analysis, participants with missing values on any covariate were excluded (Table 1 legend c).

**TABLE 1:**
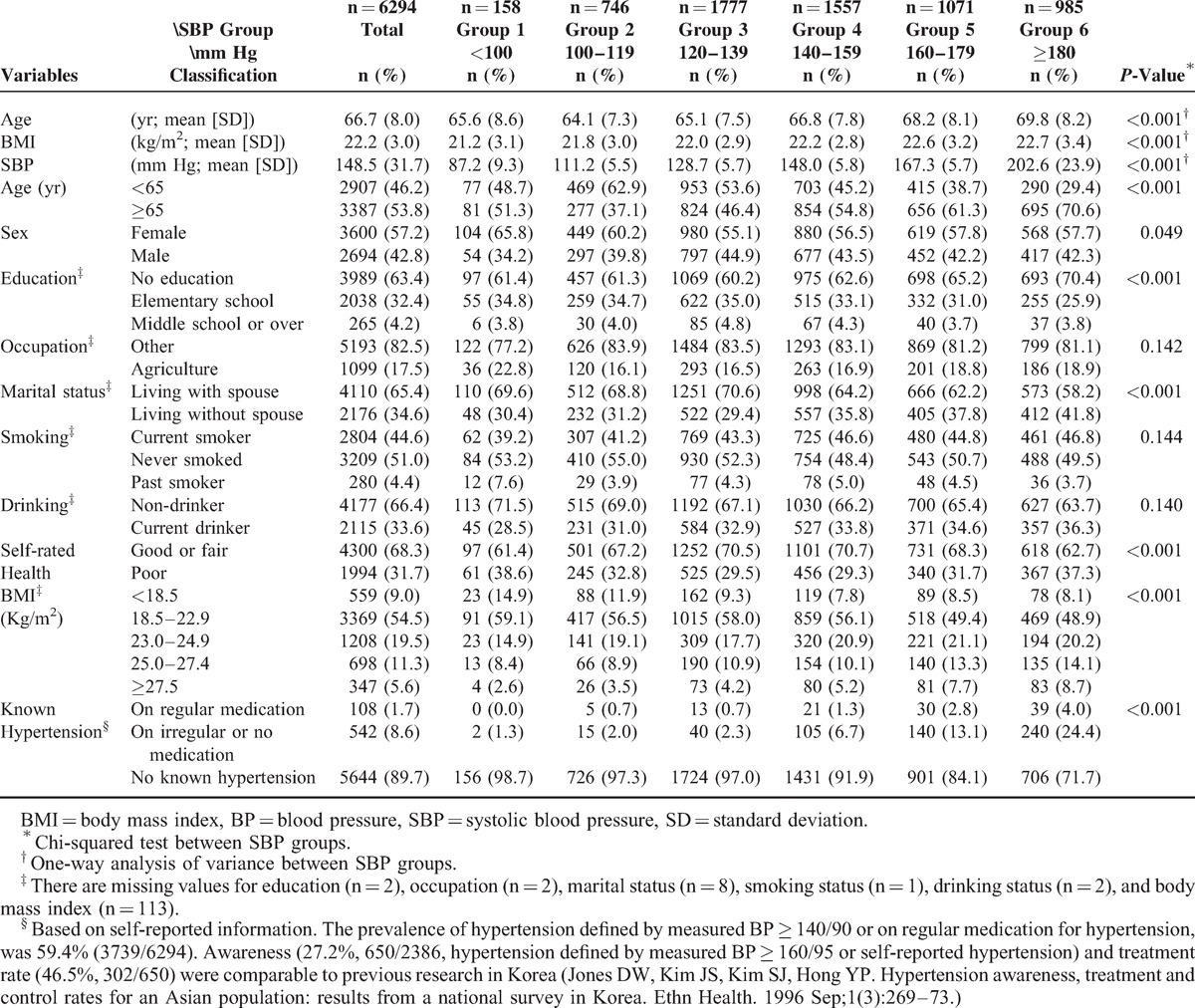
Characteristics of the Korean Elderly by SBP Group (n = 6294)

A stratified analysis was performed according to age at entry (years; ≥65, <65) to examine whether the association varies by the age group.^1,19^ Since participants who had a disease or health problem at entry might have had low BP, an analysis was implemented in which those (n = 1073) whose follow-up ended before December 31, 1989 (with less than 4.8 years of follow-up) were excluded.^11^ Additional analysis was done after excluding those (n = 810) with known vascular diseases (including hypertension, stroke, and heart diseases), or those (n = 1994) with poor self-rated health. Further analysis was done after adjustment for diastolic BP and pulse rate. These additional analyses served as a sensitivity analysis.

Two-sided *P*-values were calculated and the statistical significance level was set at 0.05. All statistical analyses were performed using SAS version 9.4 (SAS Institute, Cary, NC, USA).

## RESULTS

The total follow-up person-years numbered 90,073. Among the 4654 deaths during the 23.8 years of follow-up, 1062 participants died of vascular diseases. The average (SD) age of the participants was 66.7 (8.0) years at enrolment. Age and the proportion of poor self-rated health had a J-curve association with SBP, while BMI and the proportion of those with known hypertension increased across increasing SBP groups. Occupation, smoking status, and drinking status were not different between SBP groups (Table 1). Except for the lowest SBP (below 100 mm Hg) group, increasing SBP increased the risk of deaths from all-cause and vascular diseases including stroke and ischemic heart diseases (Table S1, http://links.lww.com/MD/A114).

**FIGURE 1:**
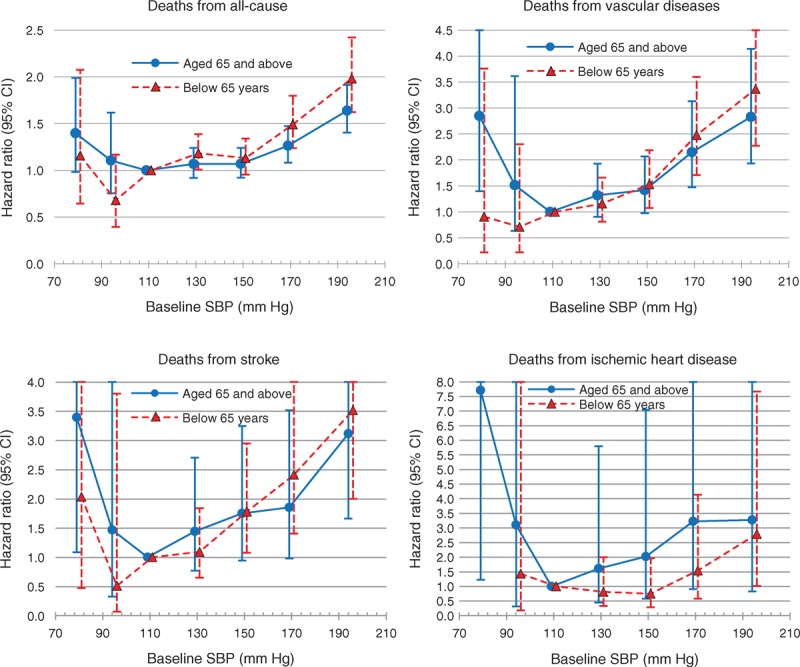
Multivariates-adjusted^a^ hazard ratio for mortality according to age group among the Korean elderly during 1985 to 2008 by 7 categories of systolic blood pressure (SBP) (mm Hg; <90, 90–99, 100–119 [Reference], 120–139, 140–159, 160–179, ≥180). The midpoint of each SBP category was used as a representative value of each category, except for the both ends (80 and 196) of SBP categories in which median was used as a representative. Death from vascular diseases (I00–I99), stroke (I60–I64), and ischemic heart diseases (I20–I25) was defined by the ICD-10. ^a^Adjusted for age at entry, sex, known hypertension (based on self-reported information), smoking status, drinking status, occupation, education, marital status, self-reported health, and body mass index. No death from ischemic heart disease was observed in the lowest SBP group among participants below 65 years.

In the stratified multivariates-adjusted analysis by age group, the lowest SBP group had an elevated adjusted hazard ratio (aHR) for mortality from vascular diseases (*P* = 0.013), based on 16 deaths, including stroke (*P* = 0.088) and ischemic heart diseases (*P* = 0.048) among participants of 65 years or above compared to those with an SBP of 100 to 119 mm Hg, while the lowest SBP was not associated with vascular mortality among people below 65 years (Table 2, Figure S1, http://links.lww.com/MD/A114). Other than for the lowest SBP group, the association between SBP and the risk of deaths from all-cause and vascular diseases did not differ by age group, while the association of high SBP with mortality was slightly stronger among participants below 65 years than it was among the elderly of 65 years or above (Table 2, Figure S1, http://links.lww.com/MD/A114). This J-curve (or U-curve) association between SBP with mortality, especially from vascular diseases, among the elderly was strengthened, when SBP was categorized into 7 groups (Figure 1, Table S2, http://links.lww.com/MD/A114), while the risk of death was the lowest for SBP of around 90 mm Hg among people below 65. The J-curve (or U-curve) association among the elderly was not weakened by additional adjustment for diastolic BP and pulse rate (Figure S2, http://links.lww.com/MD/A114).

**TABLE 2:**
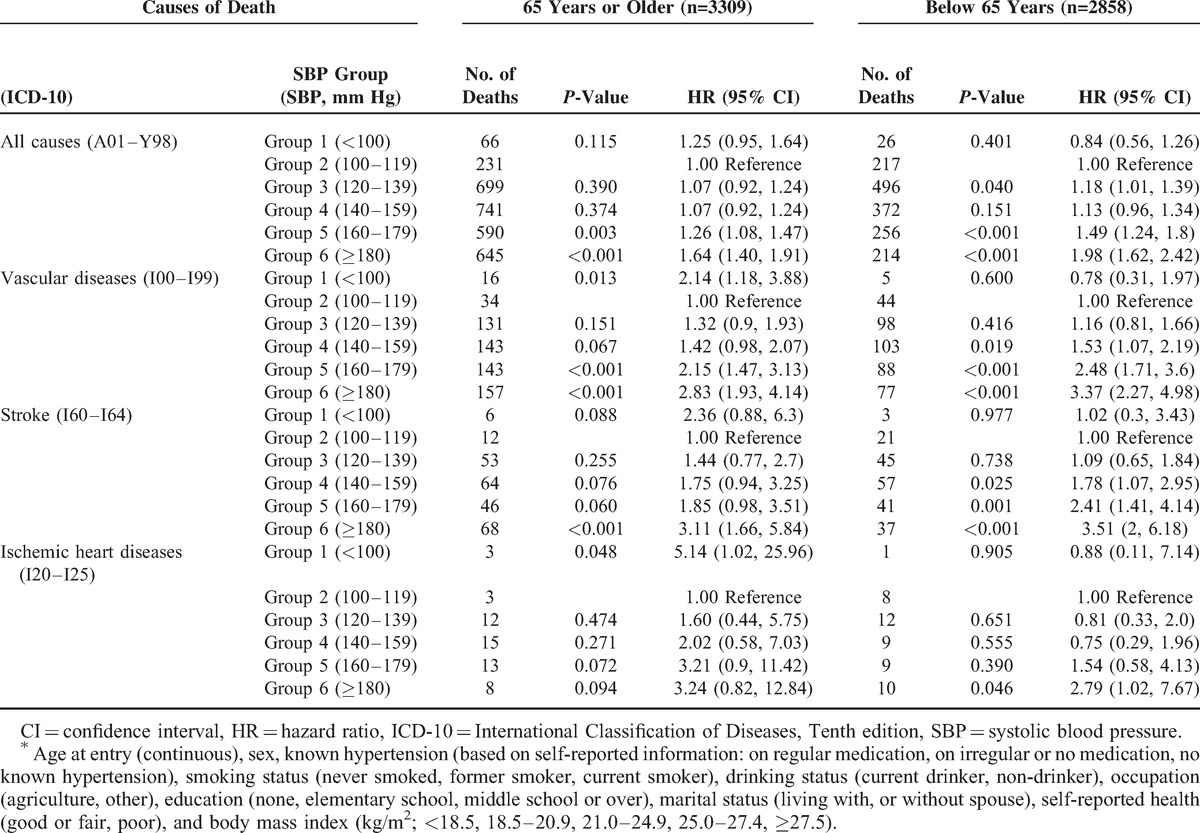
Numbers of Deaths and Adjusted^∗^ Hazard Ratio for Mortality by Age Group Among the Korean Elderly During 1985 to 2008

When the analyses were restricted to cover only survivors as of January 1, 1990, participants with no known vascular diseases at enrolment, or those with good or fair self-rated health, the results generally were not different from the main analysis (Tables S3–S5, http://links.lww.com/MD/A114).

## DISCUSSION

This study showed that a SBP below 100 mm Hg increased the risk of mortality, especially vascular mortality including stroke and ischemic heart diseases, and that furthermore, the association of SBP with the vascular mortality may be a J-curve in those aged 65 years or older, while increasing SBP nearly monotonically increased the mortality from all-cause and vascular diseases in people below 65 years.

### Reverse Causality Between Low SBP and Vascular Mortality

Since the lowest SBP group was the thinnest and had the poorest self-rated health among the SBP groups in our study, the possibility of reverse causality (ie, the suggestion that low BP may not be an independent risk factor but could be an epiphenomenon related to concurrent frailty, poor health status, or chronic diseases leading to increasing mortality) should be addressed.^11,20^ In the current study, the association of low SBP with all-cause mortality was weak and was also not significant after adjustment for potential confounders. The J-curve association with vascular mortality was not changed after adjusting for BMI and the self-rated health status. After censoring the first 4.8 years of follow-up data, the association with all-cause mortality flattened,^11^ while the J-curve association with vascular mortality in the elderly was unchanged. When those with known vascular diseases were excluded from analysis, the J-curve association was sustained. When those with poor self-rated health were excluded, the J-curve association of SBP with mortality from total vascular diseases and ischemic heart diseases (but not with stroke mortality), was maintained. Given that neither the lowest SBP nor the highest SBP was associated with cancer mortality (Table S6, http://links.lww.com/MD/A114), the hypothesis of malignancy-related BP changing mechanism may not be applicable to our study participants. Therefore, in the current study, there was no clear evidence of the reverse causality pattern that could have been hypothesized. Nevertheless, because detailed information on fragility-related health variables and medical examination was not collected and controlled for, the possibility of reverse causality cannot be completely ruled out.

Meanwhile, among those (n = 158) with SBP below 100 mm Hg, 5 participants self-reported pre-existing vascular diseases (no atrial fibrillation or valvular diseases were reported), 5 reported cancer, and 12 reported respiratory diseases. No endocrine disease (including diabetes) was reported among this group. In the mid 1980s in Korea (at study enrolment), diabetes and ischemic heart diseases were rarely a concern.

### Association Between SBP and Vascular Mortality

Except for SBP below 100 mm Hg, this study showed that increasing SBP was directly related to increasing vascular mortality with no sign of threshold, which is in line with the results of previous cohort studies.^1^ In recent studies, including many secondary analyses of data from clinical trials, a J- or U-shape association between SBP, and morbidity and mortality from vascular diseases has been reported among people with vascular diseases^2–7^ and diabetes.^8,9,21^ With few exceptions,^10,11^ for the most part these J- or U-shape associations of SBP with vascular diseases have seldom been reported in observational studies among the general population. However, previous prospective studies have mainly focused on SBP of 115 mm Hg or above and as such the association between low SBP below 100 mm Hg and vascular mortality has rarely been examined in previous research.^1^ Further research is needed to confirm this J-curve association.

The stronger association of low SBP with mortality from ischemic heart diseases rather than that with stroke mortality was in accordance with previous results among people with vascular diseases.^20^ Meanwhile, since the J-curve association of BP with mortality was observed relatively frequently in the older elderly,^19,22,23^ we analyzed data among the elderly aged 75 years or older (n = 1015) and found that the association of the lowest SBP with vascular mortality (I00–I99) was stronger (aHR = 3.7, 95% CI = 1.2–11.6, *P* = 0.02) than was true in the main analysis addressing those aged 65 years or above.

Our results showed that the risk of all-cause mortality in people with SBP of 120 to 139 mm Hg or stage 1 hypertension (140–159 mm Hg) was not particularly higher than it was in those with SBP of 100 to 119 mm Hg, especially among those aged 65 or above. These results generally concur with the recently published guideline that the therapeutic goal for SBP among the elderly should be below 150 mm Hg, rather than the previously proposed below 140 mm Hg.^24^ However, since nearly monotonic increase in vascular mortality by increasing SBP was found in this study (except for those with SBP below 100 mm Hg among the elderly), managing BP and the recommendation to lower BP through non-intensive methods such as lifestyle modification could still be beneficial for lowering the risk of vascular diseases even among those with SBP of 120 to 139 mm Hg.^25^

### Strengths and Limitations of the Study

The prospective design and nearly complete long-term follow-up using national mortality data form the principle strengths of our study. However, this study also has several limitations. First, BP was measured only once. However, analysis of data from 1 measurement should not overestimate the true hazard ratios considering the regression dilution bias.^26^ Second, since the number of participants and deaths was small due to various subgroup analyses, especially in the lowest SBP group, this may have decreased the statistical power of some analyses. However, when the lowest group further divided into 2 groups (thus, SBP was categorized into 7 group), J-curve association between SBP and vascular mortality in the elderly was consistent and even stronger than was shown in the analysis addressing 6 categories of SBP. Nonetheless, the possibility of the elevated risks in the lowest SBP group having been the result of chance alone cannot be completely ruled out due to small numbers of death. Third, the follow-up of death records was different in the 1985 to 1991 and 1992 to 2008 periods. However, only 32.5% of deaths occurred during 1985 to 1991, and even when deaths during the first 5 years were excluded, the results were similar to those in the main analysis. Fourth, the validity of the diagnosis listed on death certificates was not examined separately. Since any misclassification on the causes of death was most likely non-differential according to BP, the authors do not consider that this substantially overestimates the hazard ratios. Fifth, information on some important cardiometabolic risk factors such as blood glucose and lipid profiles were not collected and not adjusted for in this study. However, these cardiometabolic risk factors were closely related with BMI,^27^ and BMI was included in the study. Sixth, our study participants were mostly farmers from rural communities and they were very thin compared to Western populations. Thus, there may be a limitation in terms of the generalizability and some of our results may not be applied to other populations who are more obese and have a sedentary life style.^15^

## CONCLUSION

In general population under 65, the norm of “the lower, the better” is most likely applicable to SBP, and low SBP down to 90 mm Hg can be considered a sign of good health. On the contrary, in the elderly aged 65 and above, this study shows that a SBP below 100 mm Hg may increase vascular mortality. Further research is necessary to confirm the J-curve association and the causality between low SBP and vascular mortality in elderly in the community. However, our results suggest that elderly populations with low SBP should be treated with caution, since low SBP may increase vascular mortality, or (if the reverse causality hypothesis is confirmed to be true in the future) they may have unidentified underlying conditions that lead to vascular mortality. Meanwhile, this study shows that both in SBP of ≥100 mm Hg among the elderly, and in SBP down to 90 mm Hg among people below 65, increasing SBP nearly monotonically increased the vascular mortality. Therefore, these results also suggest that lowering BP through lifestyle modification may be beneficial even among those with SBP of 120 to 139 mm Hg, although lowering the SBP to below 120 mm Hg may not be a therapeutic goal in treatment for people with hypertension.
